# Porous Rod-like NiTiO_3_-BiOBr Heterojunctions with Highly Improved Visible-Light Photocatalytic Performance

**DOI:** 10.3390/ma16145033

**Published:** 2023-07-17

**Authors:** Kaiyue Sun, Mengchao Li, Hualei Zhou, Xiaohui Ma, Wenjun Li

**Affiliations:** Beijing Key Laboratory for Science and Application of Functional Molecular and Crystalline Materials, Department of Chemistry and Chemical Engineering, University of Science and Technology Beijing, Beijing 100083, China; sunkaiyue12345@163.com (K.S.); limengchao2020ks@163.com (M.L.); 18813010169@163.com (X.M.); wjli_ustb@163.com (W.L.)

**Keywords:** photocatalysis, heterojunction, porous heterostructure, NiTiO_3_, BiOBr

## Abstract

NiTiO_3_-BiOBr heterostructured photocatalysts were constructed via precipitation, calcination and hydrothermal treatments. Various characterizations demonstrated that BiOBr nanosheets were decorated on NiTiO_3_ nanoparticals, forming porous rod-like heterojunctions. Compared with independent NiTiO_3_ and BiOBr, the composites with optimal BiOBr content presented highly improved visible-light photocatalytic efficiency. The degradation rates of Rhodamine B (RhB) and tetracycline (TC) reached 96.6% in 1.5 h (100% in 2 h) and 73.5% in 3 h, which are 6.61 and 1.53 times those of NiTiO_3_, respectively. The result is an improved photocatalytic behavior from the formation of heterojunctions with a large interface area, which significantly promoted the separation of photogenerated carriers and strengthened the visible-light absorption. Based on the free radical capture experiments and band position analysis, the photodegradation mechanism of type-II heterojunction was deduced. This study provides a new way to fabricate highly efficient NiTiO_3_-based photocatalysts for degrading certain organics.

## 1. Introduction

Currently, water pollution has become an increasingly serious environmental problem. Organic pollutants in wastewater discharged from factories or hospitals are usually hazardous to humans and difficult to remove [1,2]. Solar-driven photocatalytic technology has been deemed an environmentally friendly and effective technology employed for solving problems [3,4]. Many photocatalysts have been confirmed to be able to degrade organic pollutants, including colored dyes and uncolored organics with stable structures [5], but their photocatalytic efficiency has not been satisfactory for practical needs yet.

The perovskite oxide, NiTiO_3_, has recently attracted large interest as a photocatalyst for degrading toxic pollutants due to its visible-light harvesting ability (*E*_g_ = ~2.3 eV), good electronic transportability and high stability [6,7,8,9]. Nevertheless, the photocatalytic activity of single NiTiO_3_ is inhibited by severe recombination of photogenerated carriers and narrow light absorption range [10]. One of the most effective methods to overcome the problems is combining NiTiO_3_ with other semiconductors with matched band structures to form various heterojunctions [11,12]. Among them, the staggered type-II heterojunction could not only notably accelerate the separation of the carriers but also extend/enhance the light absorption range. For instance, Shi et al. [11] fabricated Type II junctions between 2D Cu_2_WS_4_ nanosheets and 1D NiTiO_3_ nanofibers, which exhibited excellent degrading ability for RhB and TC because of improved visible-light adsorption, rapid separation of photogenerated carriers and large specific surface area. Wang et al. fabricated type-II junctions composed of 3D NiTiO_3_ nanorods and 2D MoS_2_ nanosheets, which also notably inhibited the recombination of electrons and holes and presented high photocatalytic H_2_ production [12]. Consequently, constructing efficient type-II heterojunctions is an ideal path to improving the photocatalytic performance of NiTiO_3_.

BiOBr is an efficient photocatalyst for degrading organic pollutants because of its strong oxidizing ability, lamellar structure and high chemical stability [13,14]. However, it suffers from the rapid combination of photoinduced carriers and poor visible-light absorption (E_g_ ≈ 2.7 eV) [15]. Therefore, BiOBr was often employed to construct heterojunctions with the visible-light-responsive semiconductor to enhance the solar photocatalytic property of the composites such as BiOBr-Bi_2_WO_6_ [16], BiOBr/Bi_2_MoO_6_ [17] and WS_2_/BiOBr [18], profiting from the efficient separation of photo-indued carriers and improved visible-light absorption efficiency. Supposed that BiOBr and NiTiO_3_ have matched band structures, NiTiO_3_-BiOBr heterojunctions would be expected to overcome their own shortcomings and exhibit high photocatalytic activity.

In addition, the photocatalytic behavior of the heterojunction also depends on the reasonable construction of the heterostructure, where there should be a large interface area and specific surface area for providing abundant active sites [19]. Consequently, employing the porous NiTiO_3_ rods as supporters to in-situ grow the BiOBr nanosheets, the rod-like porous NiTiO_3_-BiOBr binary heterojunctions were fabricated through precipitation, calcinations and hydrothermal methods for the first time in this study. Rhodamine B (RhB) and tetracycline (TC) were separately employed as objects of photodegradation to evaluate the performance of the composites. As a result, the composites presented highly improved photocatalytic performances compared to the single components. The photocatalyst undoubtedly exhibited high stability. Furthermore, a feasible photocatalytic mechanism was deduced based on free radical capture, EPR tests and band position measurements. This study would present a new way to construct stable NiTiO_3_-based photocatalysts for degrading certain organics.

## 2. Experiments

### 2.1. Synthesis of NiTiO_3_

NiTiO_3_ was synthesized according to the method reported by Qu et al. [20], where 2.48 g of nickel acetate (Ni(CH_3_COO)_2_·4H_2_O) was dissolved into 60 mL of ethylene glycol (EG) under stirring, and then 3.4 mL of tetra-n-butyl titanate (Ti(OC_4_H_9_)_4_) was added dropwise in sequence. During this process, light blue precipitates were produced, which gradually increased in quantity. The reaction continued for 1 h. Subsequently, the precipitates were collected, washed with deionized water and ethanol in turn, and dried at 80 °C. The resulting product was roasted in the air at 600 °C for 2 h to attain yellow NiTiO_3_ samples.

### 2.2. Synthesis of NiTiO_3_-BiOBr Composites

The NiTiO_3_-BiOBr composites were prepared by hydrothermal treatment. First, 0.1 g NiTiO_3_ was added to 30 mL of deionized water under stirring to achieve a homogeneous suspension. Second, 10 mL of EG containing an appropriate proportion of Bi(NO_3_)_3_·5H_2_O was added dropwise with stirring and then continuously stirred for an extra 30 min. Subsequently, a certain volume of 1 mg·mL^−1^ KBr solution was also added to it and fully mixed. The resulted mixture was placed into a Teflon-lined stainless autoclave (100 mL) and kept heating at 180 °C for 12 h. As a result, the product was obtained by centrifugation, washed and dried as described above. The obtained products were named NiTiO_3_-BiOBr(x), in which x signifies the designed weight percentage of BiOBr in the composite (5 wt%, 10 wt%, 15 wt% and 20 wt%, respectively). As a control experiment, a single BiOBr was synthesized with the same procedure as above without the addition of a NiTiO_3_ sample.

### 2.3. Characterization

X-ray diffraction (XRD) spectra were employed to detect the crystalline properties of the products and performed on the XRD instrument (D/MAX-RB, Rigaku, Japan). A scanning electron microscope (SEM) equipped with an energy-dispersive X-ray (EDX) spectrometer (SU8010, Hitachi, Japan) was applied to determine the morphologies and elemental distribution on the surface of samples. A transmission electron microscope (TEM, F-20, FEI, USA) was employed to analyze the internal morphology of samples by observing TEM and high-resolution TEM (HRTEM) images. Pore structure was characterized using an instrument from Quantchrome NOVA 4200e (USA). To study the chemical components and oxidation states of elements in the materials, X-ray photoelectron spectra (XPS) were tested on an XPS instrument (EscaLab 250Xi, Thermo, USA). UV-visible diffuse reflectance spectra (UV-vis DRS) were applied to explore the light absorption of samples and carried out on a UV-vis spectrophotometer (T9s, Persee, China). To detect the separation efficiency of photogenerated carriers, the photoluminescence (PL) spectra were tested on a fluorescence spectrophotometer (F-7000, Hitachi, Japan). To determine the main free radicals in the photocatalytic system, an electron paramagnetic resonance (EPR) spectrometer (JES-FA200, JEOL, Japan) was employed to obtain the EPR spectra. An electrochemical workstation (5060F, RST, China) with a three-electrode cell system was applied to attain the Mott–Schottky plots to determine the flat band potentials of pristine semiconductors. A saturated calomel electrode (SCE) and Pt filament were employed as the reference and auxiliary electrodes, respectively. A certain amount of the sample was ultrasonically dispersed in the mixed solution of ethanol and Nafion and then coated into a circular trough on the ITO conductive glass, which was dried and used as the working electrode.

### 2.4. Photocatalytic Activity Experiments

The photocatalytic properties of the samples were evaluated by degrading RhB (20 mg·L^−1^) and TC (40 mg·L^−1^). A 400 W xenon lamp with a filter (>420 nm) was used as a light source. Typically, a 30 mg sample was added to 30 mL of an aqueous solution containing the organic pollutant under stirring. The suspension was kept in the dark for 60 min to reach the adsorption-desorption balance. After the lamp was turned on, 3.0 mL of the suspension was taken out and centrifuged at regular intervals (30 min for RhB and TC). The concentrations © of RhB and TC were measured at 554 nm and 357 nm, respectively, on the T9s spectrophotometer. The value of *C*_t_/*C*_0_ could signify degradation efficiency. Here, *C*_0_ and *C*_t_ mean the concentration at the initial time and a certain time, respectively.

## 3. Results and Discussion

### 3.1. XRD Analysis

**Figure 1:**
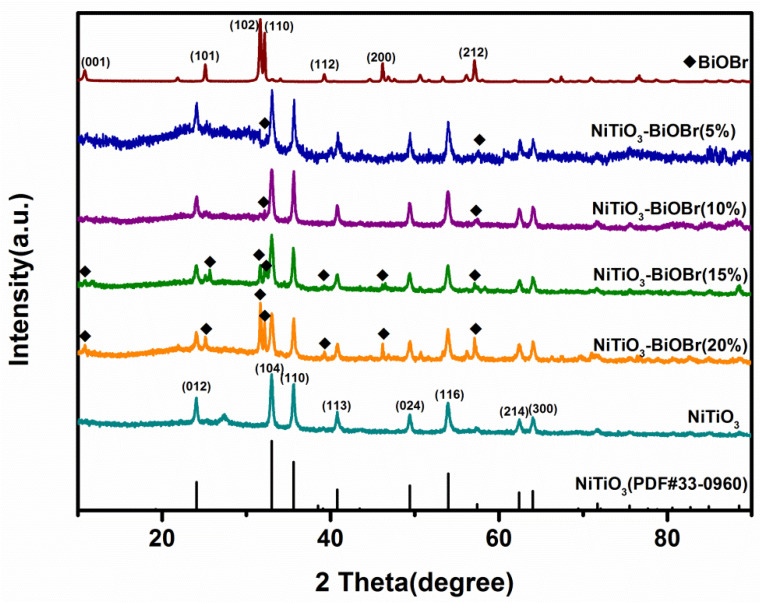
XRD patterns of the NiTiO_3_, BiOBr and NiTiO_3_-BiOBr composites.

The XRD spectra of the samples are shown in Figure 1. The pattern of single NiTiO_3_ is well in line with the standard pattern (JCPDS 33-0960). The characteristic peaks at 24.13°, 33.09°, 35.66°, 40.85°, 49.45°, 54.02°, 62.45° and 64.07° correspond to crystalline planes (012), (104), (110), (113), (024), (116), (214) and (300), respectively. In comparison, the patterns of NiTiO_3_-BiOBr composites clearly show all these peaks. Besides, the peaks at 10.90°, 25.16°, 31.70°, 32.22°, 39.38°, 46.21° and 57.12° indexed to planes (001), (101), (102), (110), (112), (200) and (212) of BiOBr (JCPDS 09-0393), respectively, could be easily observed on the patterns of the composites with high BiOBr content (15% and 20%). With the decrease in BiOBr content, the intensity of these peaks gradually decreases or becomes invisible. These results suggest the successful synthesis of the NiTiO_3_-BiOBr composite.

### 3.2. SEM and TEM Analysis

**Figure 2:**
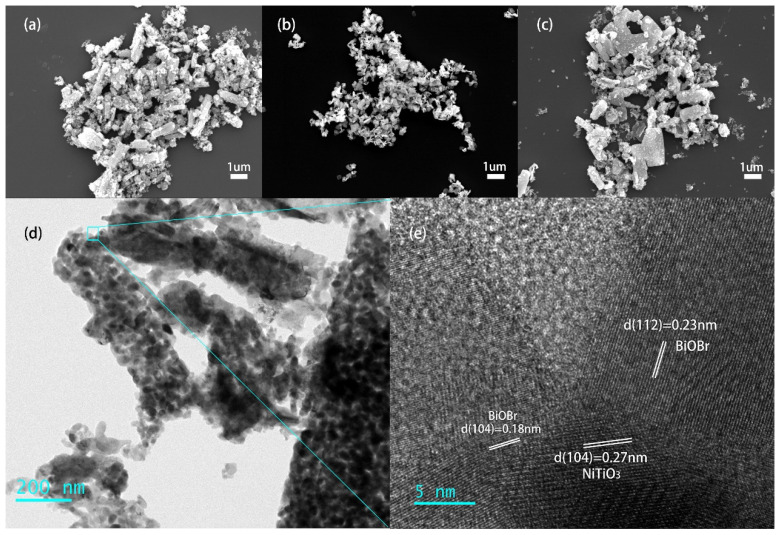
SEM pictures of (**a**) NiTiO_3_, (**b**) BiOBr, and (**c**) NiTiO_3_-BiOBr (15%) composites; (**d**) TEM image of NiTiO_3_-BiOBr (15%); (**e**) HRTEM image of NiTiO_3_-BiOBr (15%).

Taking the NiTiO_3_-BiOBr (15%) composites as an example, the morphology of the prepared samples could be determined by SEM, TEM and HRTEM images. NiTiO_3_ shows a rod-like shape with aspect ratio of ~3–5 (Figure 2a). It is also noted that a small number of irregular nanoparticles accumulates around the rods, which could be prompted by the growth of crystals during the roasting process [20]. Independent BiOBr is circular flakes with 150–300 nm in diameter (Figure 2b). The morphology of NiTiO_3_ does not significantly change after combining with BiOBr (Figure 2c). From the TEM image (Figure 2d), it can be seen that the rod-like composite is made of many tiny nanoparticles and presents a porous structure, which should have a large specific surface area and interface area. In the HRTEM image (Figure 2e), the lattice spacing of 0.27 nm is attributed to the (104) lattice planes of NiTiO_3_, and the lattice spacings of 0.23 and 0.18 nm correspond to the (112) and (104) planes of BiOBr sheets, respectively. This verifies the formation of the NiTiO_3_-BiOBr heterojunction.

**Figure 3:**
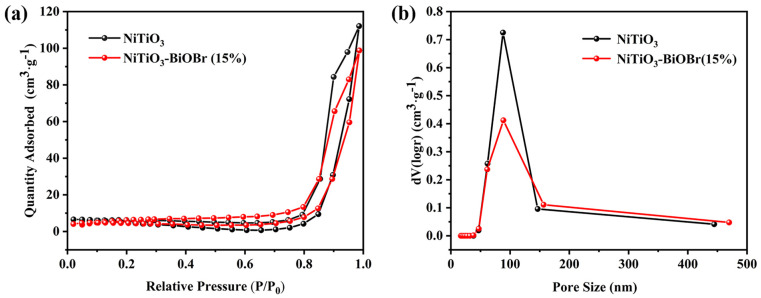
The N_2_ adsorption isotherms (**a**) and pore distributions (**b**) of NiTiO_3_ and NiTiO_3_-BiOBr (15%).

To illustrate the pore structure, N_2_ adsorption isotherms and pore distributions of NiTiO_3_ and NiTiO_3_-BiOBr (15%) are depicted in Figure 3a,b. Obviously, they belong to meso- and macro-pore structures with pore sizes of 50–150 nm, which leads to their large specific surface areas (43.2 m^2^∙g^−1^ for NiTiO_3_, 33.0 m^2^∙g^−1^ for the composite). The smaller surface area of the composite should result from the deposition of BiOBr, which blocked the partial pore of the NiTiO_3_ rod. These results indicate that the composite has many abundant active sites and large interface area, which would be very beneficial for the photocatalytic process.

### 3.3. XPS Analysis

**Figure 4:**
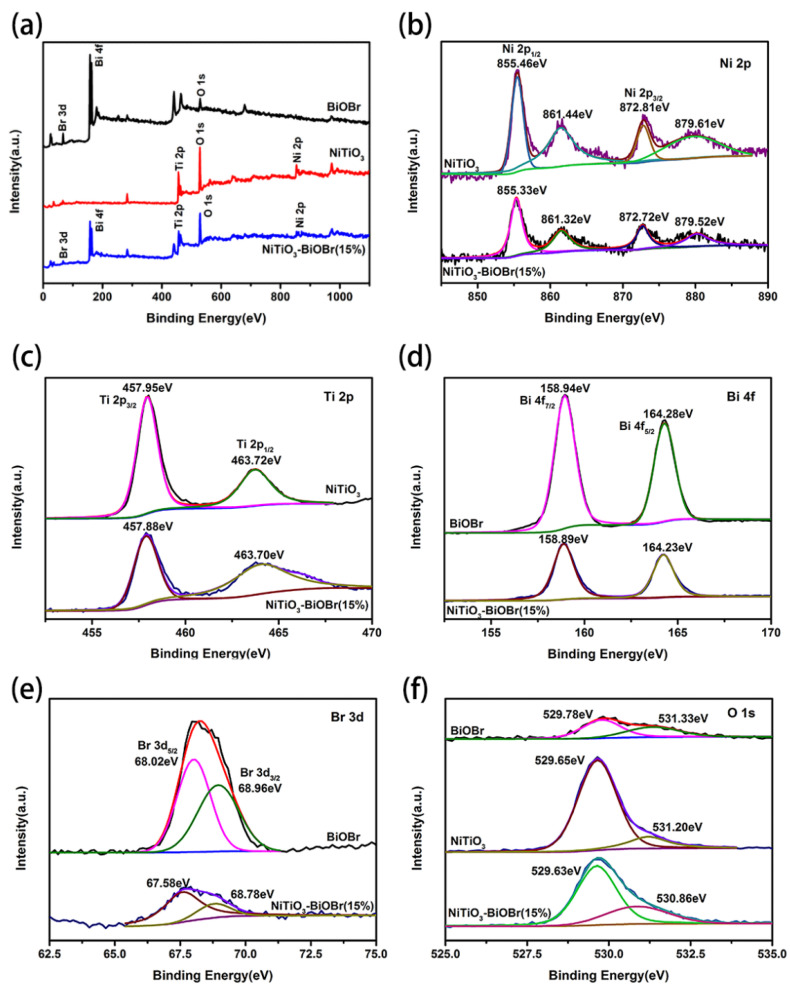
XPS analysis portraying NiTiO_3_, BiOBr and NiTiO_3_-BiOBr (15%): (**a**) Survey; (**b**) Ni 2p; (**c**) Ti 2p; (**d**) Bi 4f; (**e**) Br 3d; (**f**) O 1s.

The chemical composition and elemental oxidation states of the composites were further examined by XPS. The characteristic peaks of Ni, Ti, Bi, O and Br elements could be obviously detected on the full survey spectra of NiTiO_3_-BiOBr (15%) (Figure 4a), indicating that these elements coexist in the binary composites. The spectrum of Ni 2p (Figure 4b) was fitted into two groups of peaks separately for both NiTiO_3_ and binary composites, where the strong peaks at 855.33 and 872.72 eV are related to Ni 2p_3/2_ and Ni 2p_1/2_ of Ni^2+^, respectively, and the other two weak peaks at 861.32 and 879.52 eV should be attributed to their satellite peaks [21]. Two obvious peaks of Ti 2p at 457.88 and 463.70 eV are assigned to the Ti 2p_3/2_ and Ti 2p_1/2_ of Ti^4+^ in NiTiO_3_, respectively (Figure 4c) [21]. In Figure 4d,e, the peaks at 158.89 and 164.23 eV correspond to the Bi 4f_7/2_ and Bi 4f_5/2_ of Bi^3+^, respectively, and the peaks at 67.58 and 68.78 eV ascribe to Br 3d_5/2_ and Br 3d_3/2_ of Br^−^, separately [22,23], indicating the existence of the BiOBr component. For the O 1s spectra in Figure 4f, the peaks at 529.78 and 531.33 eV attribute to the O^2−^ of Bi-O bonds and adsorbed oxygen on the spectrum of single BiOBr, respectively [16], while the peaks at 529.65 and 531.20 eV designate the lattice O^2−^ in NiTiO_3_ and the surface-adsorbed oxygen on the spectrum of NiTiO_3_, respectively [24]. In comparison, the peak at 529.63 eV on the spectrum of the binary composites should be lattice O^2−^ in NiTiO_3_ and BiOBr, while the peak at 530.86 eV could belong to the adsorbed oxygen. Apparently, the results further suggest the successful preparation of the NiTiO_3_-BiOBr heterojunction.

### 3.4. Photocatalytic Properties

**Figure 5:**
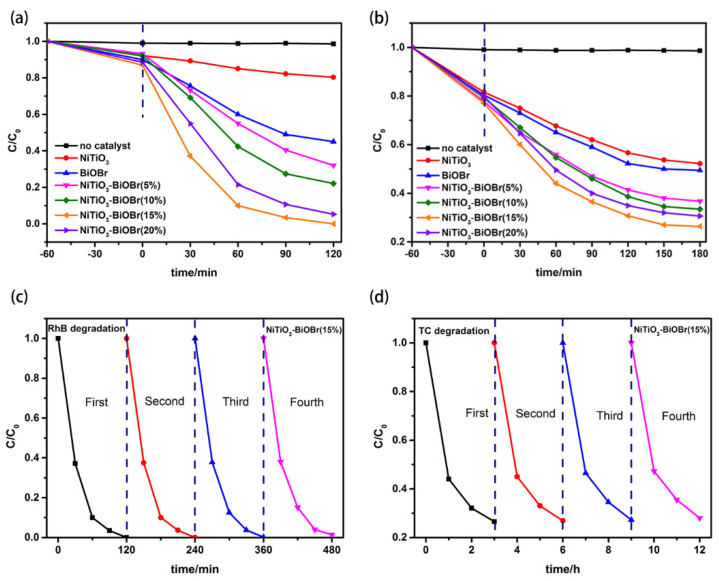
Photocatalytic degradation of prepared photocatalysts in degrading RhB (**a**) and TC (**b**); cycling tests of degrading RhB (**c**) and TC (**d**).

As shown in Figure 5a, only 14.6% and 49.0% of RhB within 1.5 h were degraded under visible-light irradiation over pristine NiTiO_3_ and BiOBr, respectively. All binary materials, comparatively, exhibit higher degradation abilities for RhB. Among them, NiTiO_3_-BiOBr (15%) presents the highest efficiency, i.e., 96.6% within 1.5 h (100% within 120 min), which is 6.61 times that of pure NiTiO_3_. This indicates that the photocatalytic activity of NiTiO_3_ in RhB degradation is amazingly boosted after coupling with an appropriate quantity of BiOBr, and the constructed NiTiO_3_-BiOBr heterojunction is highly efficient. Compared with other reported NiTiO_3_-based photocatalysts for degrading RhB, the NiTiO_3_-BiOBr photocatalyst presents notably high degradation efficiency in 90 min even if high concentration of RhB was used in this work (Table 1). The photodegradation of TC over the materials was further tested. As shown in Figure 5b, the composites also exhibit much higher activity in degrading TC than pristine NiTiO_3_ and BiOBr. The NiTiO_3_-BiOBr (15%) composite also has the highest photodegradation rate (73.5%) within 3 h, which is far higher than NiTiO_3_.

Cycling experiments over NiTiO_3_-BiOBr (15%) for RhB and TC degradation were conducted to evaluate the stability of the materials. From Figure 5c,d, it is observed that the photocatalytic rate is basically unchanged after four repeated experiments, no matter what is degraded. Therefore, the stability of the binary composites is sufficient for the practical needs of photocatalytic systems.

### 3.5. Photocatalytic Mechanism Discussion

#### 3.5.1. UV-Vis DRS Analysis

**Figure 6:**
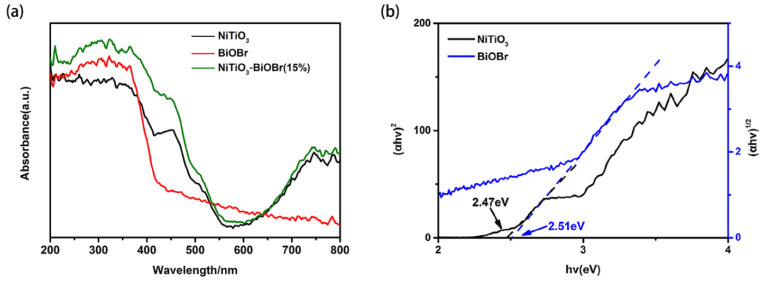
(**a**) UV-Vis DRS of NiTiO_3_, BiOBr and NiTiO_3_-BiOBr (15%) samples and (**b**) Tauc’s plots of NiTiO_3_ and BiOBr.

The UV-Vis DRS of the samples is employed to analyze their optical absorption. As shown in Figure 6a, NiTiO_3_ exhibits absorption ability in both the ultraviolet and visible light ranges. An obvious absorption peak is observed near 450 nm. According to the literature [11,20,21], crystal field splitting of the Ni-O octahedron in NiTiO_3_ crystal causes the 3d^8^ orbits of Ni^2+^ to split up into two sub-bands, and the Ni^2+^/Ti^4+^ charge-transfer could lead to the formation of two adsorption peaks at ~450 and ~510 nm (not clear), respectively. In addition, NiTiO_3_ also has light response above 600 nm due to the spin-allowing d-d transitions of Ni^2+^ [30]. In contrast, BiOBr mainly absorbs ultraviolet light and has an absorption edge of ~430 nm. By coupling them together, the composites present a notably strong visible-light absorption characteristic compared to pristine NiTiO_3_ and BiOBr.

The band gaps of the two pristine samples were determined based on the equation: (αh*ν*)^2/n^ = h*ν* − *E*_g_, where α, h, *ν*, h*ν* and *E*_g_ represent absorbance coefficient, plank constant, light frequency, irradiation energy and band gap energy, respectively [31]. Here, n is 1 for NiTiO_3_ (direct bandgap) [26] and 4 for BiOBr (indirect bandgap) [32]. Therefore, the *E*_g_ of NiTiO_3_ and BiOBr was separately determined to be 2.47 and 2.51 eV, respectively (Figure 6b).

#### 3.5.2. PL Emission Spectra

**Figure 7:**
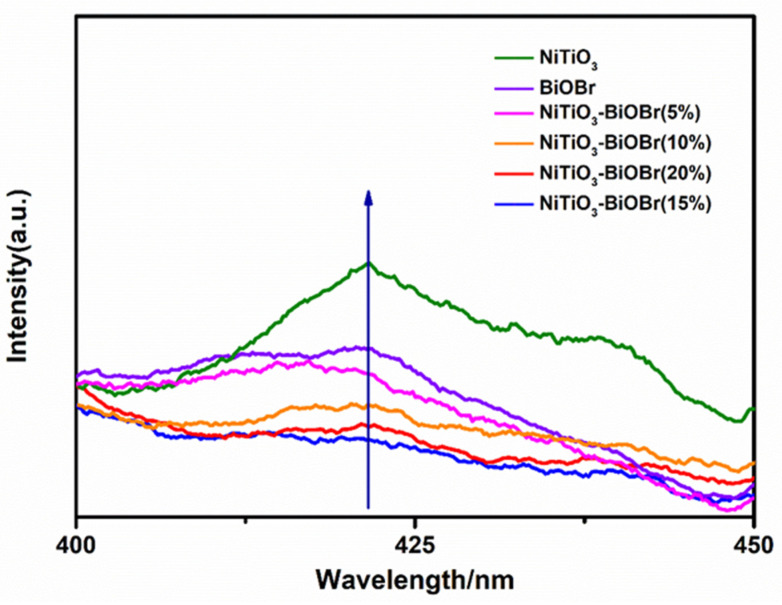
PL spectra of the samples at 280 nm of the light excitation wavelength (the arrow means the direction in which the peak values increase).

The function of the heterojunctions in inhibiting the recombination of photogenerated carriers can be revealed by PL spectra. In Figure 7, the composites present much lower peak intensities than single NiTiO_3_ and BiOBr, indicating that the formation of heterojunction greatly inhibited the recombination of photogenerated carriers and prompted the separation of the carriers. Among them, NiTiO_3_-BiOBr (15%) presents the lowest peak intensity, implying its highest separation efficiency of carriers, which corresponds to its best photocatalytic performance.

#### 3.5.3. Free Radical Capture Tests

**Figure 8:**
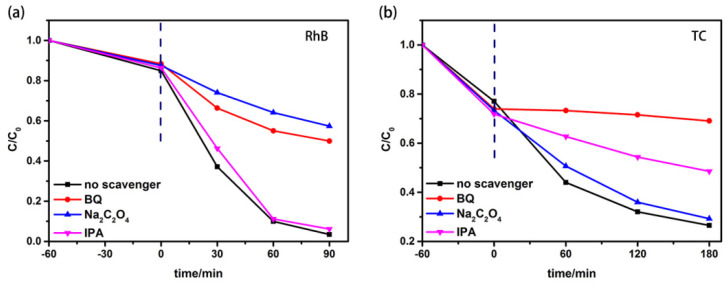
Degradation of RhB (**a**) and TC (**b**) over NiTiO_3_-BiOBr (15%) with different scavengers under visible-light irradiation.

Hydroxyl radicals (·OH), superoxide radicals (·O_2_^−^) and holes (h^+^) are usually responsible for the degradation of organics. Free radical capture tests were performed for the NiTiO_3_-BiOBr (15%) composite to investigate the degradation mechanisms of RhB and TC. Na_2_C_2_O_4_ (10 mM), isopropanol (IPA, 10 mM) and benzoquinone (BQ, 1 mM) act as scavengers for h^+^, ·OH and ·O_2_^−^, respectively. It is seen from Figure 8a that the photodegradation efficiency of RhB over NiTiO_3_-BiOBr (15%) mildly decreases after adding IPA but significantly decreases after the addition of Na_2_C_2_O_4_ or BQ, implying that h^+^ and ·O_2_^−^ are the main free radicals, while ·OH has little contribution to the RhB degradation. For TC (Figure 8b), the degradation drastically decreases to near zero after adding BQ and also clearly declines when IPA is added, while there is little change after adding Na_2_C_2_O_4_. This demonstrates that ·O_2_^−^ and ·OH play key roles in the following order: ·O_2_^−^>·OH, while h^+^ hardly contributed to the degradation of TC, possibly due to its relatively low oxidability.

**Figure 9:**
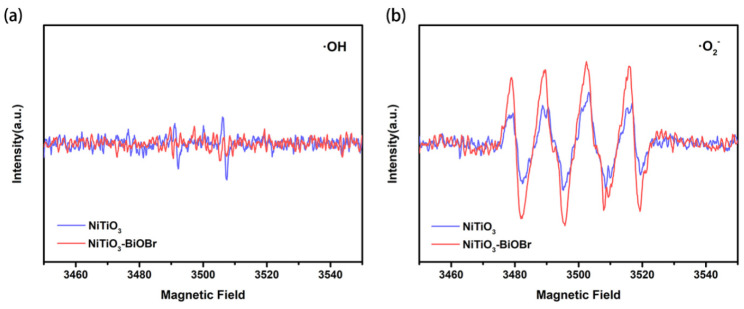
EPR spectra of (**a**) DMPO-·OH and (**b**)·O_2_^−^ over NiTO_3_ and NiTiO_3_-BiOBr composite with visible-light irradiation.

EPR measurements were performed to further explore the change of free radicals with the formation of heterojunctions. In Figure 9a, the ·OH signal in the NiTiO_3_-BiOBr composite system is hardly observed, while a weak signal could be detected in the single NiTiO_3_ system, indicating that the heterojunction does not promote or is adverse to the formation of ·OH. In contrast, the DMPO-·O_2_^−^ signals can be explicitly detected. Additionally, the composite system exhibits a stronger signal than the single NiTiO_3_ system (Figure 9b). This suggests that the heterojunction facilitates ·O_2_^−^ production, which is consistent with the results of the capture tests and show that ·O_2_^−^ plays a key role in the photocatalytic process.

#### 3.5.4. Band Position Determination

**Figure 10:**
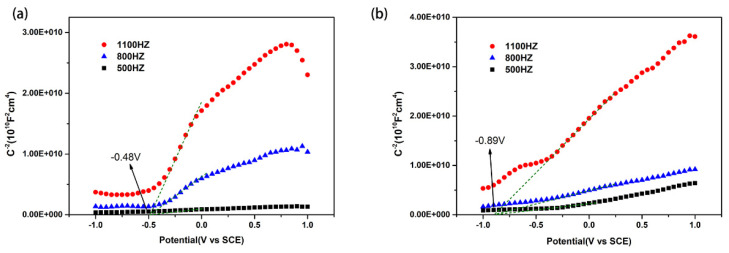
Mott–Schottky plots of (**a**) NiTiO_3_ and (**b**) BiOBr.

The band positions of NiTiO_3_ and BiOBr were determined by both Mott–Schottky (MS) plots and their band gap energies. As shown in Figure 10, both NiTiO_3_ and BiOBr belong to n-type semiconductors, as their MS plots exhibit positive slopes. The flat band energy (*E*_fb_) of NiTiO_3_ and BiOBr could be determined as −0.48 eV and −0.89 eV (νs. SCE), respectively, based on their intersections of the X-axis. The conduction band (CB) energy is usually ~0.1 eV more negative than *E*_fb_ for n-type semiconductors [33]. Consequently, the *E*_CB_ of NiTiO_3_ and BiOBr are identified as −0.34 eV and −0.75 eV vs. NHE, respectively, according to the equation: NHE = 0.24 + SCE. Based on the *E*_g_ of NiTiO_3_ (2.47 eV) and BiOBr (2.51 eV), the *E*_VB_ of NiTiO_3_ and BiOBr are 2.13 eV and 1.76 eV (*E*_VB_ = *E*_CB_ − *E*_g_), respectively.

### 3.6. Photocatalytic Mechanism of NiTiO_3_-BiOBr Photocatalysts

**Figure 11:**
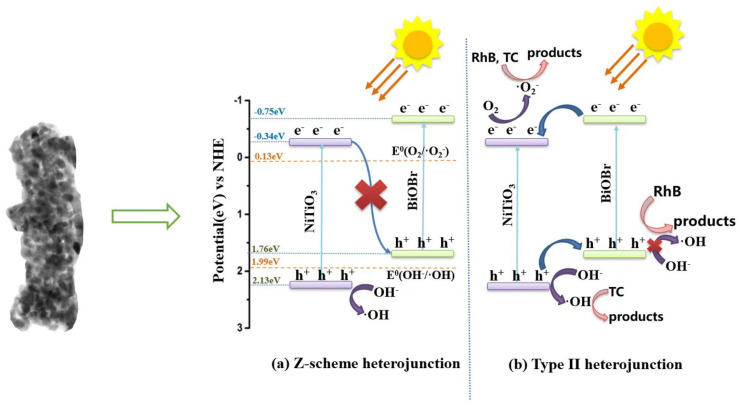
Proposed charge transfer pathway on the interface of heterojunction and photocatalytic mechanism of the NiTiO_3_-BiOBr composites in degrading organic pollutants.

Based on the above analysis, the transfer pathway of photoexcited carriers on the interface of the NiTiO_3_-BiOBr heterojunction was discussed to clarify the photocatalytic degradation mechanism. As shown in Figure 11, electrons (e^−^) on the VB of both NiTiO_3_ and BiOBr would separately attain the corresponding light energy to jump to their CB under visible-light irradiation, leaving holes (h^+^) on their VB. According to the CB and VB positions of the two semiconductors, the photo-induced carrier migration could follow the S-scheme transfer pathway (Figure 11a) [34,35] or type II transfer pathway (Figure 11b) [11,12]. Here, if the carriers follow the former, the e^−^ on the CB of NiTiO_3_ would combine with the h^+^ on the VB of BiOBr, thus inhibiting the recombination of e^−^ on CB of BiOBr and h^+^ on the VB of NiTiO_3_ and remaining their inherent redox capacity. This means that accumulating h^+^ on the VB of NiTiO_3_ in the composites could oxidize OH^−^ to generate more ·OH than that in the single NiTiO_3_. However, it is conflicted with the experimental results of the EPR (Figure 9a). Consequently, the S-scheme transfer pathway is excluded, and the conventional type II transfer pathway is proposed.

As illustrated in Figure 11b, the potential level difference between BiOBr and NiTiO_3_ would lead to the transfer of e^−^ from BiOBr to NiTiO_3_ and the transfer of h^+^ in the opposite direction, resulting in the highly efficient separation of the carriers. The e^−^ accumulated on the CB of NiTiO_3_ would reduce the adsorbed oxygen to generate a large quantity of ·O_2_^−^ due to their more negative potential than *E*^0^(O_2_/·O_2_^−^) (0.13 eV vs. NHE) [36], efficiently degrading RhB and TC. The accumulating h^+^ on the VB of BiOBr could directly oxidize RhB. Nevertheless, they could not oxidize OH^−^ to ·OH according to their lower potential than *E*^0^ (·OH/OH^−^) (1.99 eV vs. NHE) [24], which is well in line with the EPR results. Consequently, the composites exhibited notably improved photodegradation efficiency for RhB and TC due to the high separation of photogenerated carriers benefiting from the type II heterojunctions with large interface areas. Additionally, the strengthened visible light absorption of the composites also causes an improvement in their photocatalytic efficiency.

## 4. Conclusions

In this study, a series of novel binary NiTiO_3_-BiOBr materials were synthesized via precipitation, calcination and hydrothermal methods in turn. As shown by various characterizations, BiOBr nanosheets were decorated on the NiTiO_3_ nanoparticals, forming porous rod-like NiTiO_3_-BiOBr heterojunctions with large interface area. The visible-light photodegradation capacity of the binary composites was significantly boosted compared with single NiTiO_3_ and BiOBr. The optimum composite degraded 96.6% of RhB within 1.5 h (100% in 2 h) and 73.5% of TC within 3 h, which are 6.61 and 1.53 times those of NiTiO_3_, respectively. In addition, it exhibited excellent photostability. The UV-Vis DRS spectroscopy and PL analysis indicate that the NiTiO_3_-BiOBr heterojunctions strengthened visible-light absorption and notably facilitated the separation of photogenerated carriers, resulting in high photocatalytic activity. Considering the results of the free radical capture tests, EPR measurements and band position analysis, a feasible photodegradation mechanism of type-II heterojunction was proposed. This work provided a strategy for fabricating highly efficient NiTiO_3_-based photocatalysts for degrading certain organic objects.

## Figures and Tables

**Table 1:** Comparison in the photodegradation performance for RhB among NiTiO_3_-based photocatalysts.

Photocatalysts	C_0_/mg·L^−1^	Time/min	Dosage of Catalyst/g·L^−1^	Efficiency/%	Refs.
NiTiO_3_-Bi_4_NbO_8_Cl	5	90	1.0	~50	[24]
NiTiO_3_-GO	10	90	0.2	~90	[25]
NiTiO_3_-TiO_2_	-	90	1.0	~62	[26]
NiTiO_3_-Bi_2_MoO_6_	10	90	0.6	~92	[27]
NiTiO_3_-α-Fe_2_O_3_	3	90	0.1	~75	[28]
NiTiO_3_-ZnO	5 ppm	120	0.5	95	[29]
NiTiO_3_-BiOBr	20	90	1.0	96.6	This work

## Data Availability

The authors confirm that the data supporting the findings of this study are available within the article.
